# Identification and characterization of NF1 and non-NF1 congenital pseudarthrosis of the tibia based on germline *NF1* variants: genetic and clinical analysis of 75 patients

**DOI:** 10.1186/s13023-019-1196-0

**Published:** 2019-09-18

**Authors:** Guanghui Zhu, Yu Zheng, Yaoxi Liu, An Yan, Zhengmao Hu, Yongjia Yang, Shiting Xiang, Liping Li, Weijian Chen, Yu Peng, Nanbert Zhong, Haibo Mei

**Affiliations:** 1 Department of Pediatric Orthopaedics, Hunan Children’s Hospital, The Pediatric Academy of the University of South China, 86# Ziyuan Road, Changsha, Hunan Province 410007 People’s Republic of China; 2 Pediatrics Research Institute of Hunan Province, Hunan Children’s Hospital, 86 Ziyuan Road, Changsha, Hunan Province People’s Republic of China; 3 Center for Medical Genetics, School of Life Sciences, Central South University, 110 Xiangya Road, Changsha, Hunan Province People’s Republic of China; 4 Pathology Department, Hunan Children’s Hospital, 86 Ziyuan Road, Changsha, Hunan Province People’s Republic of China; 5 New York State Institute for Basic Research in Developmental Disabilities, Staten Island, NY USA

**Keywords:** Neurofibromatosis 1, Whole exome sequencing, Genomic variation, Genotype, Phenotype

## Abstract

**Background:**

Congenital pseudarthrosis of the tibia (CPT) is a rare disease. Some patients present neurofibromatosis type 1 (NF1), while some others do not manifest NF1 (non-NF1). The etiology of CPT, particularly non-NF1 CPT, is not well understood. Here we screened germline variants of 75 CPT cases, including 55 NF1 and 20 non-NF1. Clinical data were classified and analyzed based on *NF1* gene variations to investigate the genotype-phenotype relations of the two types of patients.

**Results:**

Using whole-exome sequencing and Multiplex Ligation-Dependent Probe Amplification, 44 out of 55 NF1 CPT patients (80.0%) were identified as carrying pathogenic variants of the *NF1* gene. Twenty-five variants were novel; 53.5% of variants were de novo, and a higher proportion of their carriers presented bone fractures compared to inherited variant carriers. No *NF1* pathogenic variants were found in all 20 non-NF1 patients. Clinical features comparing NF1 CPT to non-NF1 CPT did not show significant differences in bowing or fracture onset, lateralization, tissue pathogenical results, abnormality of the proximal tibial epiphysis, and follow-up tibial union after surgery. A considerably higher proportion of non-NF1 patients have cystic lesion (Crawford type III) and used braces after surgery.

**Conclusions:**

We analyzed a large cohort of non-NF1 and NF1 CPT patients and provided a new perspective for genotype-phenotype features related to germline *NF1* variants. Non-NF1 CPT in general had similar clinical features of the tibia as NF1 CPT. Germline *NF1* pathogenic variants could differentiate NF1 from non-NF1 CPT but could not explain the CPT heterogeneity of NF1 patients. Our results suggested that non-NF1 CPT was probably not caused by germline *NF1* pathogenic variants. In addition to *NF1*, other genetic variants could also contribute to CPT pathogenesis. Our findings would facilitate the interpretation of *NF1* pathogenic variants in CPT genetic counseling.

**Supplementary information:**

The online version of this article (10.1186/s13023-019-1196-0) contains supplementary material, which is available to authorized users.

## Background

Congenital pseudarthrosis of the tibia (CPT, HP: 0009736) is a rare disease characterized by either pseudarthrosis in early life or pathological fractures of the anterolateral part of the tibia presented bowing, narrowing of the medullary canal, or a cyst [1–3]. The prevalence of CPT is approximately 1 in 140,000 births [4, 5]. The treatment of CPT remains challenging and the long-term outcome of surgery is poor [6, 7]. Currently, the etiology of CPT has not been completely understood. It remains one of the most puzzle conditions in pediatric orthopedics worldwide.

CPT was previously reported to be closely related to neurofibromatosis type 1 (NF1 [OMIM: 162200]) [1, 5, 6]. About 84.0% of all CPT patients have NF1 according to a recent review [8]. NF1 is a common autosomal dominant genetic disorder affecting multi-system including skeletal and neurocutaneous systems. It was reported that about 38% of NF1 manifestations resulted from skeletal abnormalities, and the primary abnormalities included long-bone dysplasia, sphenoid-wing dysplasia, and scoliosis [9]. Long-bone dysplasia typically affects the tibia and occurs in about 5% of NF1 patients [3, 10]. NF1 is fundamentally caused by loss-of-function variants in *NF1* gene [5, 11], which have complete penetrance in adults with a high degree of variability of clinical expressions [12]. *NF1* encodes neurofibromin, a tumor suppressor negatively regulating RAS proto-oncogene to prevent cell overgrowth by inhibiting Ras/MAPK signaling [13–16]. *NF1* is expressed in the endothelial cells, glial cells, immune cells, neurons, and the adrenal medulla [12]. *NF1*-deficient osteoblasts promote the activation of osteoclasts through the secretion of cytokines such as osteopontin [16, 17]. In tibial pseudarthrosis tissue of NF1 patients, mRNA and protein expression levels decrease and p44/42 MAPK (Ras-pathway) activities are upregulated [18].

The relationship between CPT and NF1 is unclear. Not all CPT patients have NF1 and only 2–4% of NF1 patients manifest CPT [10, 19]. No significant differences were found in the cells and tissues between NF1 and non-NF1 CPT, and there was an accumulation of nerve cells surround the small arteries in the thickened periosteum of both NF1 and non-NF1 CPT [20]. Both NF1 and non-NF1 CPT showed lower osteogenicity in the cultured bone marrow stromal cells from the lesion tissue [21]. However, the genetic background and pathogenesis of the two types of CPT remain unclear. The associated clinical manifestations, interventions and outcomes of this disease remain to be clarified. In this study, we included 75 CPT patients from 74 trios (55 NF1 and 20 non-NF1). We combined whole-exome sequencing (WES), Multiplex Ligation-Dependent Probe Amplification (MLPA) and comprehensive clinical data analysis to investigate the genetic background and the associated phenotypes related to germline *NF1* variants.

## Results

### *NF1* pathogenic variants were identified in 58.7% CPT cases and predominantly affected NF1 CPT

**Fig. 1:**
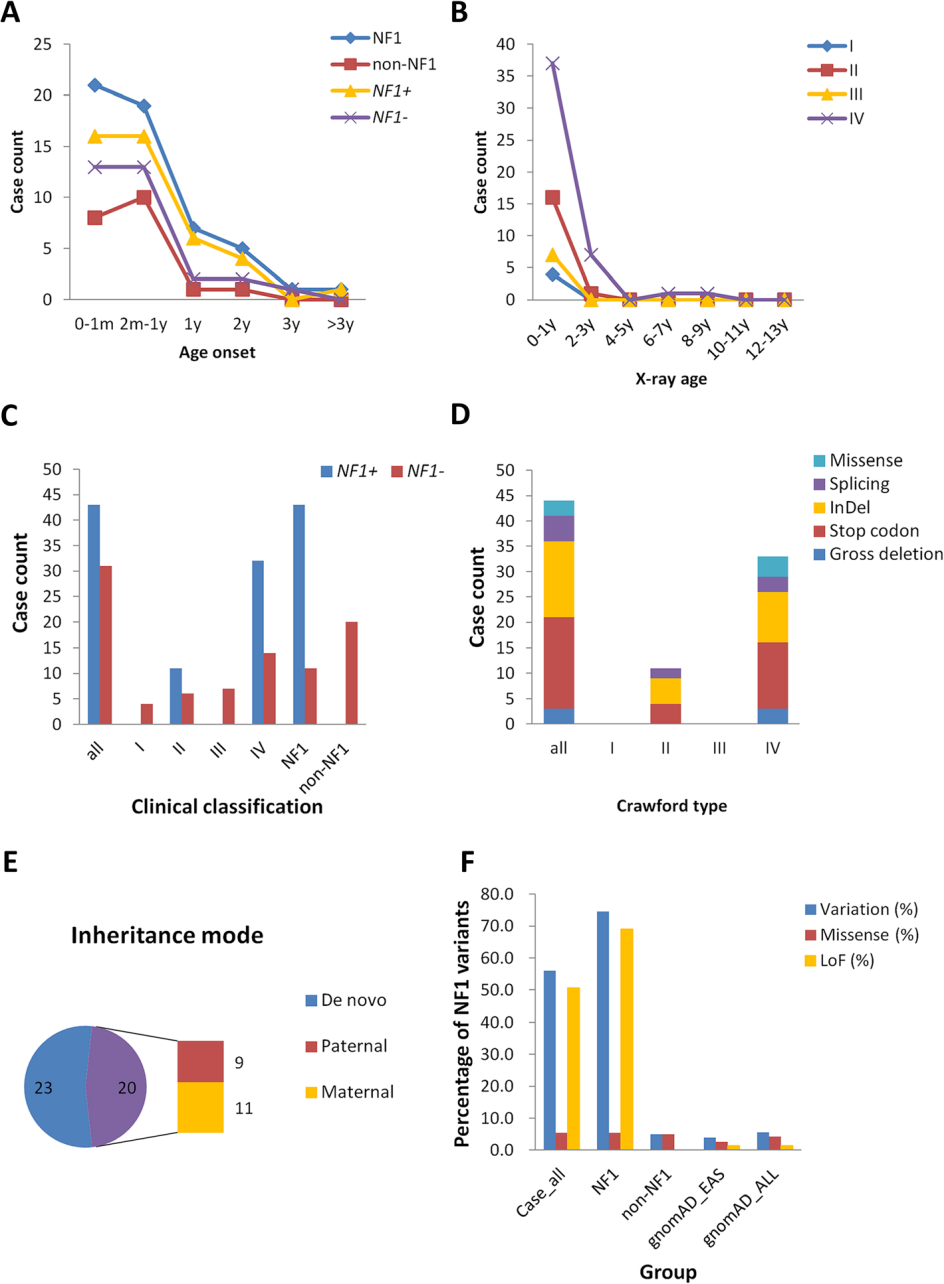
Clinical classification and *NF1* pathogenic variants identified in 75 CPT patients. **a**. The distribution of the number of cases in different onset-age in NF1 CPT patients, non-NF1 CPT patients, *NF1*^+^ (with* NF1 *pathogenic variants identified) patients, and *NF1*^−^ (no *NF1* pathogenic variants identified) patients. **b**. The distribution of the number of cases in four different Crawford types classified when CPT occurred according to age stage. y: year. **c**. The distribution of the number of *NF1*^+^ (blue bar) and *NF1*^−^ (red bar) patients in different clinical classification groups. **d**. The distribution of exonic functional effect of *NF1* pathogenic variants in different Crawford type patients. The majority variants are stop codon (blue bar), InDel (red bar) or splicing (green bar) variants, only three are missense variants (purple bar). **e**. The inheritance mode distributed in 43 CPT patients (exclude 5B) identified *NF1* pathogenic variants. De novo variants show in blue, and inherited variants show in purple which is consist of paternal mode (red bar) and maternal mode (green bar). **f**. Bar plot of the percentage of rare SNVs and InDels of the *NF1* gene in NF1 and non-NF1 CPT patients compared to gnomAD database. Nonsynonymous variants in the coding region of the *NF1* gene with MAF < 0.005 were calculated. gnomAD_EAS: East Asian population of gnomAD, gnomAD_all: all population. LoF: loss-of-function associated variants, including stop-gain, splicing changes, startlost, stoplost and InDels

**Fig. 2:**
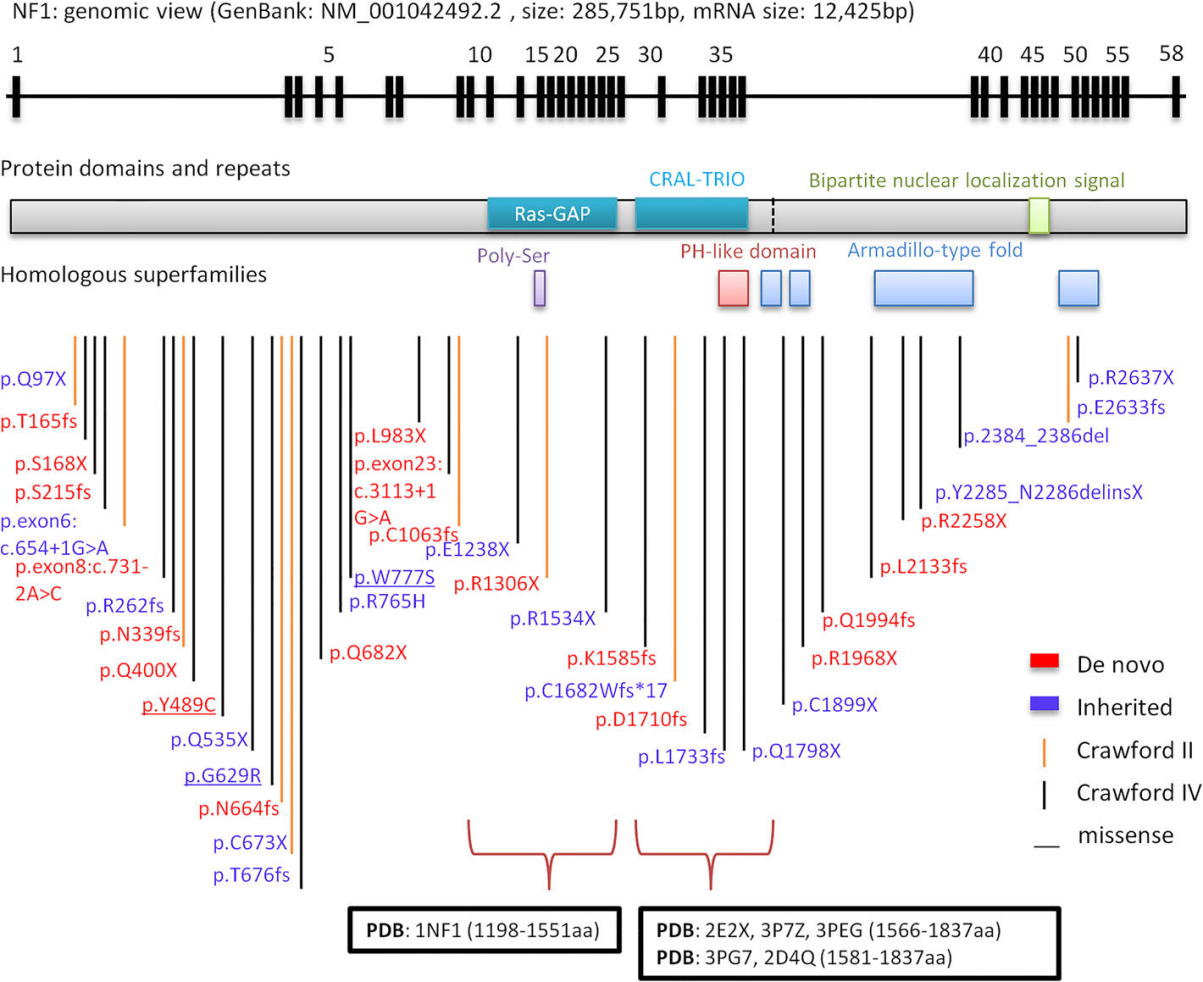
*NF1* pathogenic variants identified by WES in genomic and protein view. *NF1* pathogenic variants view from genome to protein secondary structure and domain. Genomic view: showing in the top with black bars marked as the relative position of exons from *NF1* gene transcript variant 1 (GenBank: NM_001042492.2). *NF1* pathogenic variants map: *NF1* pathogenic variants identified in this study are marked at the bottom according to the relative position of protein amino acids. *NF1* de novo variants show the amino acid change label in red color; inherited variants show in purple color. Vertical lines show variant position, and Crawford type IV shows in black color, Crawford type II shows in orange color. Protein domains and repeats, homologous superfamilies (InterPro: P21359): Ras GAP domain (1187-1557aa, glaucous bar), CRAL-TRIO lipid-binding domain (1580-1738aa, glaucous bar), Bipartite nuclear localization signal domain (2555-2571aa, green bar), Ploy-Ser domain (1352-1355aa, purple bar), PH-like domain superfamily (1727-1837aa, red bar), Armadillo-type fold superfamily (1849-1886aa, 1920-1984aa, 2200-2420aa and 2613-2676aa, blue bar). Ras GAP and CRAL-TRIO lipid binding domains with PDB structure are marked at the bottom showing amino acid positions and PDB accessions

Among NF1 CPT patients, *NF1* heterozygous pathogenic variants (Fig. 1c) were detected in 44 cases (44/55–80.0%), including 25 novel variants (Table 1). Sixteen cases had pathogenic variants that were recorded in ClinVar; these variants were seen in NF1 patients, among whom three had CPT phenotypes (Table 1). The variants included 18 stop codons, 15 InDels, 5 splice sites, 3 missense variants and 3 gross deletions (Fig. 1d, Table 1, Additional file 1: Figure S1). Out of the 44 pathogenic variants, 43 (97.7%) had damaging functional effects (loss-of-function), which were interpreted as pathogenic variants based on ACMG criteria [22]. The proportion of loss-of-function associated variants (MAF < 0.005) was dramatically higher in NF1 CPT patients than in all populations and the East Asian population in gnomAD database (74.5% vs. 1.4%) (Fig. 1f, Additional file 5: Table S2). The three missense variants (p.(Tyr489Cys), p.(Gly629Arg), and p.(Trp777Ser)) were close to N-terminus ahead of Ras GAP domain (Fig. 2). p.(Tyr489Cys) and p.(Gly629Arg) were recorded in ClinVar as pathogenic. p.(Tyr489Cys) was found to cause the downstream of 62 nt at cDNA c.1466_1527del at exon 13 and then formed a stop codon at AA 489 in five patients [23]. p.(Gly629Arg) (c.G1885A) generated a cryptic 3′ splice site that resulted in a cDNA with 1846_1886del [24]. p.(Trp777Ser) (c.G2330C) was reported in six NF1 patients, and was interpreted as likely pathogenic in ACMG and ClinVar (Table 1). The identified *NF1* pathogenic variants were located at various positions and showed high heterogeneity. Only two variants were shared by two families (44A and 45A shared p.Q400X; 37A and 75A shared c.3113 + 1G > A, Table 1). The region near the N-terminus harbored slightly more variants than the C-terminus of neurofibromin (Fig. 2). In addition, partial or entire *NF1* deletions were found in three patients (10A, 15A, 35A) (Table 1).

**Table 1:** Information of *NF1* pathogenic variants identified in 75 CPT cases

Sample ID	Exon position	Nucleotide Change^a^	Amino Acid Change^a^	ACMG Criteria	Novel / Known Variation	PMID Reported CPT
71A	exon 4	c.289C > T	p.(Gln97*)	Pathogenic	Novel	
17A	exon 5	c.499_502del	p.(Cys167Glnfs*10)	Pathogenic	ClinVar	
5A, 5B	exon 5	c.503C > G	p.(Ser168*)	Pathogenic	ClinVar	
51A	exon 6	c.643del	p.(Ser215Alafs*10)	Pathogenic	Novel	
26A	exon 6	c.654 + 1G > A		Pathogenic	Novel	
47A	exon 8	c.731-2A > C		Pathogenic	Novel	
48A	exon 8	c.786_787insTT	p.(Lys263Leufs*19)	Pathogenic	Novel	
22A	exon 9	c.1019_1020del	p.(Ser340Cysfs*12)	Pathogenic	Novel	
44A, 45A	exon 11	c.1198C > T	p.(Gln400*)	Pathogenic	Novel	
29A	exon 13	c.1466A > G	p.(Tyr489Cys)	Pathogenic	ClinVar^b^	23668869
6A	exon 14	c.1603C > T	p.(Gln535*)	Pathogenic	Novel	
52A	exon 17	c.1885G > A	p.(Gly629Arg)	Pathogenic	ClinVar	
23A	exon 17	c.1992dup	p.(Ser665Leufs*5)	Pathogenic	Novel	
36A	exon 18	c.2019C > A	p.(Cys673*)	Pathogenic	Novel	
54A	exon 18	c.2033dup	p.(Ile679Aspfs*21)	Pathogenic	Novel	
24A	exon 18	c.2044C > T	p.(Gln682*)	Pathogenic	Novel	
43A	exon 20	c.2330G > C	p.(Trp777Ser)	Likely pathogenic	ClinVar	
74A	exon 22	c.2947del	p.(Leu983*)	Pathogenic	Novel	
37A, 75A	exon 23	c.3113 + 1G > A		Pathogenic	ClinVar	
41A	exon 24	c.3187_3188insTA	p.(Cys1063Leufs*15)	Pathogenic	Novel	
18A	exon 28	c.3712G > T	p.(Glu1238*)	Pathogenic	ClinVar	
72A	exon 29	c.3916C > T	p.(Arg1306*)	Pathogenic	ClinVar	
59A	exon 35	c.4600C > T	p.(Arg1534*)	Pathogenic	ClinVar^b^	23668869
64A	exon 36	c.4756_4772del	p.(Ala1586Tyrfs*30)	Pathogenic	Novel	
27A	exon 37	c.5046delinsGGTTAC	p.(Cys1682Trpfs*18)	Pathogenic	Novel	
2A	exon 37	c.5130del	p.(Cys1711Valfs*9)	Pathogenic	Novel	
7A	exon 37	c.5199dup	p.(Glu1734Argfs*23)	Pathogenic	Novel	
31A	exon 38	c.5392C > T	p.(Gln1798*)	Pathogenic	Novel	
55A	exon 39	c.5697 T > A	p.(Cys1899*)	Pathogenic	Novel	
62A	exon 40	c.5902C > T	p.(Arg1968*)	Pathogenic	ClinVar^b^	24232412
3A	exon 40	c.5980_5983del	p.(Ala1994Lysfs*17)	Pathogenic	Novel	
39A	exon 42	c.6401_6402del	p.(Cys2134Tyrfs*8)	Pathogenic	Novel	
53A	exon 45	c.6772C > T	p.(Arg2258*)	Pathogenic	ClinVar	
1A	exon 45	c.6819 + 1_6825del		Pathogenic	Novel	
50A	exon 46	c.6854dup	p.(Tyr2285*)	Pathogenic	ClinVar	
4A	exon 48	c.7159_7164del	p.(Asn2387_Phe2388del)	Pathogenic	Clinvar^c^	
40A	exon 54	c.7898del	p.(Glu2633Glyfs*11)	Pathogenic	Novel	
56A	exon 54	c.7909C > T	p.(Arg2637*)	Pathogenic	ClinVar^b^	16773574
10A	exon 1–58	c.-383_*3522del	p.0	Pathogenic	ClinVar	
15A	exon 13–30	c.1393_4110del	p.(Ser465_Gln1370del)	Pathogenic	Novel	
35A	exon 36–58	c.4725_*3522del	p.?	Pathogenic	Novel	

^a^Position annotated based on *NF1* transcript 1 (GenBank: NM_001042492.2, GenPept: NP_001035957.1)

^b^Only one case reported having tibial pseudarthrosis

^c^Same variant position but different variant types

*PMID* PubMed ID

7

### No germline *NF1* variants were identified in non-NF1 CPT patients

No *NF1* coding region pathogenic variants were identified in 31 cases (31/75; 41.3%), including 20 non-NF1 CPT patients (100%) and 11 NF1 CPT patients (11/55; 20.0%) (Additional file 4: Table S1); thus, all non-NF1 patients had no family history of NF1 (Additional file 4: Table S1, Fig. 1c). In non-NF1 patients, the frequency of rare SNVs and InDels (MAF < 0.005) in the coding region of *NF1* gene was similar to that of general population (5% vs. 5.6%) and East Asian population in gnomAD database (5% vs.3.9%) (Additional file 5: Table S2, Fig. 1f). One non-NF1 proband (32A) was found to have a missense variant (NP_001035957.1:p.(Arg765His)) of NF1, which was reported in ClinVar (variation ID: 68313) as “uncertain significance” (same as ACMG interpretation). This variant was inherited from the patient’s father who had no NF1. It should be investigated whether this variant is associated with CPT.

### Similar clinical features in NF1 CPT and non-NF1 CPT

The clinical features of NF1 and non-NF1 CPT were analyzed, including manifestations, interventions and outcomes (Table 2, Additional file 2: Figure S2). The age of onset is mostly were below three years (72/74–97.3%), with majority showing onset in the first year (Fig. 1a, Table 2). As the individuals grow, *NF1* variants identified in each onset age showed similar proportions (Pearson correlation coefficient = 0.98, Fig. 1a) and no obvious tendency of transformation from non-NF1 CPT to NF1 CPT was observed (Fig. 1a). Overall, there were no significant differences between the two CPT types in tibia bowing or fracture onset, lateralization, pathological detection of periosteum and cortical bone, abnormality of the proximal tibial epiphysis, and the follow-up of tibia union after surgery (Table 2). For the morphological and radiological features, all patients had tibia angulation deformity. NF1 CPT and non-NF1 CPT patients showed no significant differences in preserved medullary canal (Crawford type I), narrowed medullary canal with cortical thickening and trabeculation defect (Crawford type II) and pseudarthrosis appearance (Crawford type IV). All the four types of Crawford classification showed no significant correlation with the age of affected individuals (Spearman correlation coefficient = 0.2). All tissue-available samples of pseudarthrosis showed fibrovascular tissue hyperplasia, and the majority of samples showed hyaline degeneration and thick-walled angiogenesis. In addition, a small fraction of pseudarthrosis tissues was observed as mucoid denaturation, inflammatory cell infiltration, multinuclear giant cells, or chondroid tissue (Table 2, Additional file 4**:** Table S1). Their distribution in NF1 CPT and non-NF1 CPT groups showed similar a percentage. One non-NF1 CPT sample (19A) showed pigmented granules in lesion tissue and one NF1 CPT sample (10A) showed hemosiderin granules (Additional file 4**:** Table S1).

**Table 2:** Statistical data of clinical features of 74 probands in four groups: NF1 vs. non-NF1, *NF1*^+^ vs. *NF1*^−^

Clinical Group	Features^a^	*NF1* ^*+*^	*NF1* ^−^	NF1 CPT	non-NF1 CPT	*NF1*^*+*^ %	*NF1*^*−*^ %	NF1 CPT %	non-NF1 CPT %	Fisher’s test *P* value (*NF1*^+^ vs. *NF1*^−^)	Fisher’s test *P* value (NF1 vs. non-NF1)
Total	74	43	11	54	20						
Bowing time										0.098	0.587
	<1y	32	10	42	19	82.1	90.9	84.0	95.0		
	1-3y	7	0	7	1	17.9	0.0	14.0	5.0		
	>3y	0	1	1	0	0.0	9.1	2.0	0.0		
	NA	4	0	4	0						
Crawford classification										0.004	0.001
	I	0	2	2	2	0.0	18.2	3.7	10.0		
	II	11	4	15	2	25.6	36.4	27.8	10.0		
	III	0	1	1	6	0.0	9.1	1.9	30.0		
	IV	32	4	36	10	74.4	36.4	66.7	50.0		
Fracture										0.156	0.247
	Yes	20	5	18	14	71.4	45.5	56.3	73.7		
	No	8	6	14	5	28.6	54.5	43.8	26.3		
	NA	15	0	22	1						
Fracture time										0.161	0.265
	<1y	14	4	18	8	42.4	80.0	47.4	57.1		
	1-3y	14	0	14	2	42.4	0.0	36.8	14.3		
	>3y	5	1	6	4	15.2	20.0	15.8	28.6		
	NA	10	6	16	6						
Lateralization										0.502	0.558
	Unilateral	41	10	51	20	95.3	90.9	94.4	100.0		
	Bilateral	2	1	3	0	4.7	9.1	5.6	0.0		
Brace										0.129	0.008
	Yes	34	6	40	20	79.1	54.5	74.1	100.0		
	No	9	5	14	0	20.9	45.5	25.9	0.0		
Tibial union on last followup										0.171	0.435
	Yes	36	11	47	19	83.7	100.0	87.0	95.0		
	No	7	0	7	1	16.3	0.0	13.0	5.0		
APTE										0.09	0.659
	Yes	4	0	4	2	36.4	0.0	7.4	10.0		
	No	7	11	50	18	63.6	100.0	92.6	90.0		
Pathology											
	FTH	34	5	39	15	100.0	100.0	100.0	100.0		
	HD	29	5	34	14	85.3	100.0	87.2	93.3		
	TWA	34	4	38	14	100.0	80.0	97.4	93.3		
	MD	4	3	7	1	11.8	60.0	17.9	6.7	0.032	0.419
	CTF	8	3	11	5	23.5	60.0	28.2	33.3	0.125	0.747
	ICI	3	0	3	3	8.8	0.0	7.7	20.0	1	0.331
	MGC	5	1	6	2	14.7	20.0	15.4	13.3		
	NA	9	6	15	5						

^a^y - year(s) old; *NF1*^*+*^ *NF1* pathogenic variants identified, *NF1*^−^ no *NF1* pathogenic variants identified. *NA* not available, *APTE* Abnormality of the proximal tibial epiphysis, *FTH* Fibrovascular tissue hyperplasia, *HD* hyaline degeneration, *TWA* thick-walled angiogenesis, *MD* mucoid denaturation, *CTF* chondroid tissue focally, *MGC* multinuclear giant cells, *ICI* inflammatory cell infiltration

### More non-NF1 CPT patients were Crawford type III and tend to use braces

**Fig. 3:**
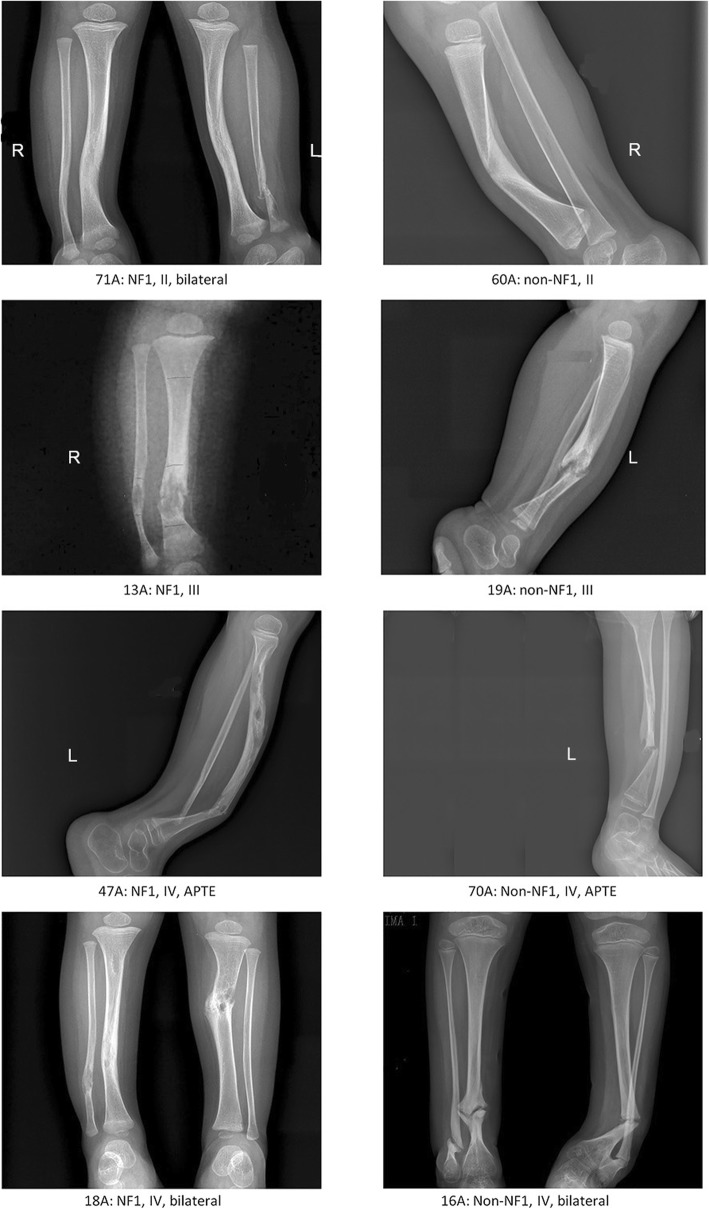
X-ray images of four NF1 CPT vs. four non-NF1 CPT patients. Four NF1 CPT patients show at the left column, and four non-NF1 CPT patients show at the right column. Case 71A (NF1) and 60A (non-NF1) are Crawford II type showing cortical thickening and narrowed medullary canal; case 13A (NF1) and 19A (non-NF1) are Crawford III type with cystic lesion; case 47A (NF1) and 70A (non-NF1) were Crawford IV type presenting pseudarthrosis and an abnormality of the proximal tibial epiphysis (APTE); case 18A (NF1) and 16A (non-NF1) are bilateral and are classified as Crawford IV type

There were two features showed significant differences. First, in Crawford classifications using X-ray, significantly more non-NF1 CPT patients had cystic lesion and were classified as Crawford III compared to NF1 CPT patients (6/20–30% vs. 1/54–1.9%, OR = 0.039, *P*-value = 0.001). However, concerning NF1 and non-NF1 CPT patients with the same Crawford type, similar morphological and radiological features were observed (Fig. 3). Second, all 20 non-NF1 CPT patients and 40 out of 54 NF1 CPT patients used brace in this study (100% vs. 74.1%, OR = 1.914, *P*-value = 0.008). This suggests that more non-NF1 CPT patients with cystic lesion but not presenting pseudarthrosis used brace during their treatment. Regarding tibia union in the last follow-up, only one non-NF1 patient did not show tibia union (union rate: 95%) and there was no union in 7 out of 54 NF1 patients (union rate: 87%).

### Bilateral pseudarthrosis were observed in all NF1 CPT patients

In our study, only three (16A, 18A, 71A) NF1 CPT patients had uncommon bilateral pseudarthrosis (Additional file 4: Table S1). They all had NF1 with more than one location showing manifested neurofibromatosis 1. No non-NF1 CPT patients had bilateral pseudarthrosis. Non-NF1 CPT is more likely to have one localized phenotype.

### Genetic heterogeneity and clinical heterogeneity based on *NF1* pathogenic variants

The evaluated *NF1* variants mostly caused loss of function. No significant correlations were found between the variant types of *NF1* and the clinical features (Fisher’s test P-value > 0.05, Additional file 6: Table S3, Additional file 3: Figure S3 A). Interestingly, two *NF1* variants were respectively shared by two unrelated patients. First, 44A and 45A shared the same de novo nonsense variant p.(Gln400*) (Table 1). However, 44A presented tibia bowing at seven-month-old with the narrowing of the medullary canal, cortical thickening, and trabeculation defect. The tissue of the patient’s lesion site showed fibrovascular tissue hyperplasia and thick-wall angiogenesis (Additional file 4: Table S1). The patient also had an abnormality of proximal tibial epiphysis while 45A did not present such features. 45A presented more serious bone atrophy with narrowing of the ends of the two fragments (named pseudarthrosis, Crawford type IV) with tibia bowing at six-month-old (Additional file 4: Table S1). His lesion site also showed partial hyaline degeneration. Second, 37A and 75A shared a de novo variant c.3113 + 1G > A (Table 1); 37A presented of the thinned medullary canal, cortical thickening and trabeculation defect (Crawford type II) after birth and reached tibial union on the last follow-up after surgery using bracing (Additional file 4: Table S1), and 75A presented pseudarthrosis (Crawford type IV) at two months old, and there was no union after surgery without brace (Additional file 4: Table S1). These findings indicate that no direct genotype-phenotype association was detected using Crawford classification and other clinical indicators.

In addition, individuals carrying the same *NF1* variant in a family did not show consistent CPT phenotype. In 20 NF1 CPT cases with family history of CPT, only one case (5A, 5%) inherited a p.Ser168* variant from the father and both patients had tibial pseudarthrosis. In contrast, no CPT manifestations were found in either father or mother of other 19 cases. In ClinVar 3460 *NF1* variants (860 benign or likely benign, 1116 pathogenic or likely pathogenic, 1441 uncertain significance, and 43 others) were reported, among which only four cases had pseudarthrosis (Table 1). Thus, no obvious CPT manifestations were closely related to variation type, inheritance mode and specific variant-position of *NF1*, suggesting that NF1 and CPT caused by *NF1* gene variants have high clinical heterogeneity.

### Over half of NF1 CPT patients had de novo pathogenic variants and frequently showed fractured bones

Twenty-three (53.5%) de novo pathogenic variants were found in 40 probands (excluding 5B in family 5) (Additional file 4: Table S1, Additional file 1: Figure S1). Since 55 CPT patients (20 non-NF1 and 35 NF1, 55/75 = 73.3%) had no family history of CPT or NF1 (Additional file 4: Table S1), the de novo variant rate might be under-evaluated. In 20 inherited CPT cases, nine variants were inherited from the father and 11 variants were inherited from the mother (Fig. 1e). Interestingly, two cases (18A, 71A) presented rare bilateral tibial pseudarthrosis and each harbored a stop-gain variant inherited from the mother. Four cases (15A, 44A, 47A, 64A) showed an abnormality of proximal tibial epiphysis all had de novo variants. Compared to inherited variants, patients harboring de novo variants showed a significantly higher rate of fracture (Additional file 6: Table S3, *P*-value = 0.000042). Other clinical features showed no much discrepancy (Additional file 3: Figure S3).

## Discussion

To our knowledge, this is the first study performing genetic and clinical analysis of *NF1* pathogenic variants between NF1 and non-NF1 CPT patients. The purpose of our study was to clarify the genetic basis and the associated clinical features related to germline *NF1* variants. Our results revealed that non-NF1 CPT with localized phenotype had no *NF1* germline pathogenic variants but generally presented similar pseudarthrosis features as NF1 CPT. *NF1* germline pathogenic variants were only identified in NF1 CPT patients who showed high clinical heterogeneity, particularly in family members carrying the same variant and presenting inconsistent tibia features. No direct genotype-phenotype correlations were found. Interestingly, significantly high proportion of non-NF1 CPT patients presented cystic lesion before bone fracture (Crawford type III) and used bracing during the treatment, while all three bilateral pseudarthrosis patients were NF1 CPT. These findings suggest that non-NF1 CPT could be a separate entity and have a different genetic cause.

CPT manifests dramatically before one year old and the age of onset is not related to the NF1-type and Crawford classification. CPT patients commonly have a high rate of fracture recurrence. Bone morphogenetic protein (BMP) in treatment has no advantages in improving initial union, and decreasing the duration between union and refracture episodes [25]. Therefore, genetic and molecular factors rather than an environmental factor are more likely contributing to CPT pathogenesis. The diversity of clinical phenotypes and *NF1* germline pathogenic variants suggest the complexity of the disease-causing mechanism of CPT. Bone formation and destruction required a balanced interplay between osteoblasts and osteoclasts. Osteoblasts can facilitate proliferation. *NF1*-deficient osteoblasts have decreased ability of proliferation and mineralization, while osteoclasts increase in the lesion site of tibial pseudarthrosis [26, 27]. In *NF1* conditional knockout mouse models with inactivation of *Nf1* in osteochondroprogenitors or the undifferentiated mesenchymal cells in the developing limbs, tibial dysplasia were also observed [28, 29]. Loss of neurofibromin hyperactivates RAS and is speculated to cause increased cell growth and survival including pigmented lesions, tumor, and skeletal defects such as tibial pseudarthrosis [15, 30, 31]. In pathological detection of pseudarthrosis tissue from NF1 CPT patients, highly cellular fibrocartilage (also known as fibrous hamartoma) was found [18, 32, 33]. Fibrous hamartoma cell lacks osteoblastic differentiation in response to BMPs [32, 34]. The lesion tissue exhibits low osteogenic ability and high osteoclastogenicity [21, 33, 35]. All our detected thickened periosteal tissues including NF1 type and non-NF1 type presented fibrous tissue hyperplasia and most had proliferating thick-wall blood vessels. This is consistent with previous studies [20]. The small arteries surrounded by nerve cells in the periosteum might inhibit the supply of nutrient to the subperiosteal bone and mesenchymal stromal cells (MSC), and thus impair the differentiation of osteoblasts [20, 36]. In a somatic variant screening of pseudarthrosis tissue in NF1 CPT, no other genes but recurring somatic variants of *NF1* were detected (sometimes termed double inactivation) [37]. Our result confirmed that *NF1* loss-of-function variant is a major factor leading to NF1 CPT.

The limitation of WES and MLPA might make some *NF1* variants undetected. For example, microdeletions, inversion, translocation or abnormal karyotype might interfere with NF1 [12, 38–40]. In addition, non-coding variants from the regulating area of *NF1* could be among the undetected genetic lesions. In addition to germline loss-of-function variants of *NF1*, somatic variants occuring in fetal development could be another potential disease-causing factor [12, 37, 39]. For non-NF1 CPT exhibiting tibial dysplasia without other NF1 features but showing similar pathological features as NF1 CPT in the lesion tissue, localized somatic mosaicism or segmental NF1 in the tibia could be present [39]. Comprehensive detection and analysis of other variants using the lesion tissue and the blood of non-NF1 CPT and NF1 CPT are needed to answer these questions.

It remains to be determined whether other modifying genes or variants might play an important role in the CPT lesion. Not all NF1 CPT were found to have loss of biallelic *NF1* in the soft proliferative pseudarthrosis tissue [37, 41, 42]. Somatic double inactivation probably is not the key disease-causing factor of the local tibial lesion. In addition, the lesion in the tibia is a rare phenotype in NF1 patients, with less than 5% of NF1 patients presenting with tibial pseudarthrosis [3, 10]. Concerning the inherited *NF1* pathogenic variants, there was a low consistency in CPT manifestation between probands and variant-positive parents having NF1. In our study, only 5A and his father harbored the same NF1 variant and both presented CPT. Finally, no *NF1* pathogenic variants were identified in non-NF1 CPT but these patients presented similar clinical features compared to NF1 CPT. Taken together, these findings implied that other genetic factors might contribute to CPT pathogenesis. It deserves to conduct other genetic or molecular screenings using either the tissue or the blood to further investigate the pathogenesis of CPT disease.

Similar to non-NF1 CPT, osteofibrous dysplasia (OFD), also known as fibroosseous steofibrous dysplasia has a benign fibroosseous lesion in the tibia of children. It is necessary to distinguish the clinical features and pathogenesis between OFD and non-NF1 CPT patients. OFD is often asymptomatic, painful, and deforming [43, 44]. According to previous studies, CPT occurs in earlier infancy or childhood and presents more severe deformity at tibia diaphysis compared to OFD [45, 46]. In addition, CPT is usually limited to the distal third of the tibia, whereas OFD might spread longitudinally to the metaphysis as the lesion progresses. For magnetic resonance and radiographic features, OFD often shows complete intramedullary extension or perilesional marrow edema with well-margined osteolytic lesions [45]. In this study, we excluded OFD according to these features in our examined non-NF1 CPT cases.

## Conclusions

We analyzed a large cohort of CPT cases, including non-NF1 CPT and NF1 CPT, by screening for germline pathogenic variants using WES and MLPA. Our results demonstrated that sharing a similar tibial manifestation as NF1 CPT, non-NF1 CPT was not related to germline NF1 pathogenic variants. Germline *NF1* pathogenic variants predominantly affected NF1 CPT, but could not explain their clinical heterogeneity in the tibia among the variant-carriers. We suggest that other genetic variations might play an important role in CPT pathogenesis.

## Methods

### Aim, design and settings

The aim of this study was to investigate variants and characterize clinical features between NF1 CPT and non-NF1 CPT patients. We screened variants using WES and MLPA in 55 NF1 CPT patients and 20 non-NF1 CPT patients, and performed genetic analysis and clinic analysis to clarify their associations resulting from *NF1* variants of the two types of patients.

The department of pediatric orthopaedics of Hunan Children’s Hospital is the largest center of CPT treatment in China. It has 68 beds and admits about 80 CPT patients every year. We receive CPT patients across the mainland of China.

### Participants

A consecutive cohort of 75 cases (55 NF1, 20 non-NF1) was enrolled in this study. Patients having osteofibrous dysplasia were excluded in this study. We collected the detailed clinical information and family history of 74 probands (provided in Additional file 4: Table S1). Peripheral blood of 74 trios was preserved. Only sample 5A (son) and sample 5B (father) came from the same family. The average age of probands was 3.8 years old (Fig. 1a, b). The youngest patient was three-month-old and the oldest patient was 13-year-old (Additional file 4: Table S1). Their average age of tibia-bowing-presence was six months. The ratio of male to female cases was 3:2. By X-ray examination performed at tibia bowing or fracture onset, there were 46 probands classified as Crawford type IV, 7 were type III, 17 were type II, 4 were type I (Additional file 4: Table S1) [47]. In total, 20 cases had one single phenotype of tibial pseudarthrosis (HP:0009736) and were clinically diagnosed as non-NF1 type (NIH, 1988) [48]. 55 cases (55/75–73.3%) accompanied multiple Cafe-au-lait spots (CAL, HP:0007565) and were diagnosed as NF1 type (NIH, 1988) [48]. In which, three cases also presented subcutaneous neurofibromas, and 15 cases had a family history of multiple CALs and subcutaneous neurofibromas. Only three patients (16A, 18A, 71A) had bilateral pseudarthrosis manifestation. Five patients (8A, 15A, 47A, 64A, 70A) presented abnormality of proximal tibial epiphysis (HP: 0010591). Biopsy of periosteum and partial cortical bone of the patients who underwent surgery was performed using H&E, and the pathological results of each patient were collected in Additional file 4: Table S1. The X-ray images of eight patients (4 NF1, 4 non-NF1) were provided in Fig. 3.

### Whole-exome sequencing and bioinformatic analysis

Genomic DNA from peripheral blood was extracted using the standard phenol-chloroform method. DNA of all 75 CPT patients was fragmented and exome was captured using the Agilent SureSelect Human All Exon V6 kit. The captured DNA was sequenced with 2 × 150 bp reads by Illumina HiSeq X Ten system (Illumina, San Diego, California, USA) following the manufacturer’s instructions. Each sample yielded over 12 Gb raw data. Over 89% (average ~ 92.9%) bases had Phred quality score > 30.

The sequenced raw reads in FastQ file format were preprocessed using Trimmomatic (version 0.33, http://www.bioinformatics.babraham.ac.uk/projects/trim_galore/) to trim low-quality bases (Phred score < 10) and adapter-contaminated ends. The polished reads whose length < 36 bp were removed to obtain the clean data. The high-quality reads were subsequently mapped to the human reference sequence (version: GRCh38) employing the alignment tool Burrows-Wheeler Aligner (BWA, Version 0.7.7) [49]. SAMtools [50] and Picard (version 1.106, https://broadinstitute.github.io/picard/) were run to remove the duplicate reads. The Genome Analysis Toolkit (GATK, version 3.1.1) [51] was applied to realign locally and recalibrate base quality scores to generate the refined bam file, and then to call single nucleotide variations (SNVs) and short insertions and deletions (InDels). The SNVs and InDels were subsequently performed functional annotation by ANNOVAR [52] and InterVar (version 20,180,118) [53]. Phenotype-based annotation was performed using Phenolyzer [54]. The SNPs and InDels with population frequency (Minor Allele Frequency, MAF) > 0.1% in gnomAD, 1000genome and ESP6500 databases were removed. We also filtered out the variants collected in our in-house database. The remaining non-benign heterozygous variants annotated by InterVar or ClinVar (version 20,180,603) in the coding or UTR regions were then kept for further analysis. We analyzed the remaining variants by calculating the number of variants and patients from the same gene one by one. The gene having the highest variation frequency was prioritized and the variants within the gene were selected for subsequent validation.

The prioritized variants of the *NF1* gene were screened in ClinVar (https://www.ncbi.nlm.nih.gov/clinvar/) and HGMD databases (public version, http://www.hgmd.cf.ac.uk) for known pathogenic records. By combining the automatically interpretation of InterVar and personalized information (such as family history, phenotype cosegregation and previous study results), the clinical classification of each variant according to ACMG criteria was further customized. Protein domains and repeats, homologous superfamilies of neurofibromin were queried from InterPro (http://www.ebi.ac.uk/interpro).

### Sequence validation with sanger

The candidate variants in *NF1* gene identified by WES were validated using Sanger method in the trios (affected probands, father and mother). PCR primers were designed using the Primer-blast program (https://www.ncbi.nlm.nih.gov/tools/primer-blast/). All the variants were validated by independent PCR amplification and DNA bidirectional sequencing performed on an ABI 3130 DNA analyzer. Segregation patterns were obtained to determine whether the variant cosegregated with the CPT phenotype in the pedigree.

### Multiplex ligation-dependent probe amplification (MLPA)

For the NF1 CPT patients unidentified *NF1* variants by WES, deletions or duplications encompassing > = 1 *NF1* exon or entire gene were detected using MLPA. We used the SALSA MLPA probe P081 NF1 mix 1 and P082 NF1 mix 2 (MRC-HOLLAND, Amsterdam, the Netherlands) to screen the DNA of peripheral blood and performed dosage analysis following the manufacturer’s instructions.

### Statistical analysis

74 CPT probands were divided into four groups: 54 of NF1 CPT, 20 of non-NF1 CPT, 43 with *NF1* pathogenic variants identified (*NF1*^+^), and 11 NF1 CPT but without *NF1* pathogenic variants identified (*NF1*^−^). Statistical analyses were performed using IBM SPSS 20.0 software (IBM SPSS, Inc., Chicago, IL). In the analysis of clinical features, Chi-square test and Fisher’s exact test were applied to compare between NF1 CPT group and non-NF1 CPT group, and between *NF1*^+^ group and *NF1*^−^ group. Odds ratio (OR) value of clinical features was calculated. All *P* values calculated were two-sided. Spearman correlation coefficient was calculated between age distribution and NF1 classification in CPT patients. Pearson correlation coefficient was calculated between the number of *NF1*^+^ patients and their age distribution.

## Supplementary information

Additional file 1: **Figure S1.** Sequencing profile of identified variants in trios by Sanger sequencing. All 41 trios had performed Sanger sequencing and this figure shows three of them. “A” in sample ID represents probands, “B” represents the proband’s father, “C” represents the proband’s mother. (TIF 560 kb)

Additional file 2: **Figure S2.** Box plot of percentage of clinical features presented in four groups of CPT patients: NF1, non-NF1, *NF1*^+^ and *NF1* ^−^ . (TIF 1261 kb)

Additional file 3: **Figure S3.** Distribution of exonic functions and inheritance mode of variants in clinical features. A. Distribution of exonic functions against clinical features. No significant *p*-value of Fisher’ test was found in each feature. B. Distribution of inheritance mode against clinical features. Fracture shows a significant difference with p-value = 4.2E-05 (Fisher’s test). (TIF 2734 kb)

Additional file 4: **Table S1.** The detailed clinical and genetic information of participated CPT cases. (XLSX 20 kb)

Additional file 5: **Table S2.** Statistics of rare variants in the coding region of the *NF1* gene in CPT patients compared to gnomAD population. (XLS 18 kb)

Additional file 6: **Table S3.** The number of patients having different *NF1* variant types distributed in evaluated clinical features. (XLS 21 kb)

## Data Availability

All data generated or analyzed during this study are included in this published article and its additional files.

## Abbreviations

CPT: Congenital pseudarthrosis of the tibia
MLPA: Multiplex Ligation-Dependent Probe Amplification
NF1 CPT: Congenital pseudarthrosis of the tibia with more than one NF1 features according to NF1 criteria. It’s classified as NF1
*NF1*^−^: CPT patients having NF1 without *NF1* pathogenic variants identified
NF1: Neurofibromatosis type 1
*NF1*^+^: CPT patients with *NF1* pathogenic variants identified
Non-NF1 CPT: Congenital pseudarthrosis of the tibia with no other NF1 features excepting tibial dysplasia according to NF1 criteria. It’s not classified as NF1
WES: Whole-exome Sequencing

## References

[1] Crawford AH (1986). Neurofibromatosis in children. Acta Orthop Scand Suppl.

[2] Hefti F, Bollini G, Dungl P, Fixsen J, Grill F, Ippolito E (2000). Congenital pseudarthrosis of the tibia: history, etiology, classification, and epidemiologic data. J Pediatr Orthop B.

[3] Stevenson DA, Birch PH, Friedman JM, Viskochil DH, Balestrazzi P, Boni S (1999). Descriptive analysis of tibial pseudarthrosis in patients with neurofibromatosis 1. Am J Med Genet.

[4] Crawford AH, Schorry EK (1999). Neurofibromatosis in children: the role of the orthopaedist. J Am Acad Orthop Surg..

[5] Vitale MG, Guha A, Skaggs DL (2002). Orthopaedic manifestations of neurofibromatosis in children: an update. Clin Orthop Relat Res.

[6] Vander Have KL, Hensinger RN, Caird M, Johnston C, Farley FA (2008). Congenital pseudarthrosis of the tibia. J Am Acad Orthop Surg.

[7] Granchi D, Devescovi V, Baglio SR, Magnani M, Donzelli O, Baldini N (2012). A regenerative approach for bone repair in congenital pseudarthrosis of the tibia associated or not associated with type 1 neurofibromatosis: correlation between laboratory findings and clinical outcome. Cytotherapy.

[8] Van Royen K, Brems H, Legius E, Lammens J, Laumen A (2016). Prevalence of neurofibromatosis type 1 in congenital pseudarthrosis of the tibia. Eur J Pediatr.

[9] Sabbagh A, Pasmant E, Imbard A, Luscan A, Soares M, Blanche H (2013). NF1 molecular characterization and neurofibromatosis type I genotype-phenotype correlation: the French experience. Hum Mutat.

[10] Friedman JM, Birch PH (1997). Type 1 neurofibromatosis: a descriptive analysis of the disorder in 1728 patients. Am J Med Genet.

[11] Knudson AG (1993). Introduction to the genetics of primary renal tumors in children. Med Pediatr Oncol.

[12] Gutmann DH, Ferner RE, Listernick RH, Korf BR, Wolters PL, Johnson KJ (2017). Neurofibromatosis type 1. Nat Rev Dis Primers.

[13] Andersen LB, Ballester R, Marchuk DA, Chang E, Gutmann DH, Saulino AM (1993). A conserved alternative splice in the von Recklinghausen neurofibromatosis (NF1) gene produces two neurofibromin isoforms, both of which have GTPase-activating protein activity. Mol Cell Biol.

[14] Ballester R, Marchuk D, Boguski M, Saulino A, Letcher R, Wigler M (1990). The NF1 locus encodes a protein functionally related to mammalian GAP and yeast IRA proteins. Cell..

[15] Sharma R, Wu X, Rhodes SD, Chen S, He Y, Yuan J (2013). Hyperactive Ras/MAPK signaling is critical for tibial nonunion fracture in neurofibromin-deficient mice. Hum Mol Genet.

[16] de la Croix NJ, Makowski AJ, Uppuganti S, Vignaux G, Ono K, Perrien DS (2014). Asfotase-alpha improves bone growth, mineralization and strength in mouse models of neurofibromatosis type-1. Nat Med.

[17] Elefteriou F, Benson MD, Sowa H, Starbuck M, Liu X, Ron D (2006). ATF4 mediation of NF1 functions in osteoblast reveals a nutritional basis for congenital skeletal dysplasiae. Cell Metab.

[18] Leskela HV, Kuorilehto T, Risteli J, Koivunen J, Nissinen M, Peltonen S (2009). Congenital pseudarthrosis of neurofibromatosis type 1: impaired osteoblast differentiation and function and altered NF1 gene expression. Bone..

[19] Young H, Hyman S, North K (2002). Neurofibromatosis 1: clinical review and exceptions to the rules. J Child Neurol.

[20] Hermanns-Sachweh B, Senderek J, Alfer J, Klosterhalfen B, Buttner R, Fuzesi L (2005). Vascular changes in the periosteum of congenital pseudarthrosis of the tibia. Pathol Res Pract.

[21] Granchi D, Devescovi V, Baglio SR, Leonardi E, Donzelli O, Magnani M (2010). Biological basis for the use of autologous bone marrow stromal cells in the treatment of congenital pseudarthrosis of the tibia. Bone..

[22] Richards S, Aziz N, Bale S, Bick D, Das S, Gastier-Foster J (2015). Standards and guidelines for the interpretation of sequence variants: a joint consensus recommendation of the American College of Medical Genetics and Genomics and the Association for Molecular Pathology. Genet Med.

[23] Messiaen LM, Callens T, Roux KJ, Mortier GR, De Paepe A, Abramowicz M (1999). Exon 10b of the NF1 gene represents a mutational hotspot and harbors a recurrent missense mutation Y489C associated with aberrant splicing. Genet Med.

[24] Xu W, Yang X, Hu X, Li S (2014). Fifty-four novel mutations in the NF1 gene and integrated analyses of the mutations that modulate splicing. Int J Mol Med.

[25] Kesireddy N, Kheireldin RK, Lu A, Cooper J, Liu J, Ebraheim NA (2018). Current treatment of congenital pseudarthrosis of the tibia: a systematic review and meta-analysis. J Pediatr Orthop B.

[26] Yang FC, Chen S, Robling AG, Yu X, Nebesio TD, Yan J (2006). Hyperactivation of p21ras and PI3K cooperate to alter murine and human neurofibromatosis type 1-haploinsufficient osteoclast functions. J Clin Invest.

[27] He Y, Rhodes SD, Chen S, Wu X, Yuan J, Yang X (2012). C-Fms signaling mediates neurofibromatosis Type-1 osteoclast gain-in-functions. PLoS One.

[28] Kolanczyk M, Kossler N, Kuhnisch J, Lavitas L, Stricker S, Wilkening U (2007). Multiple roles for neurofibromin in skeletal development and growth. Hum Mol Genet.

[29] Wang W, Nyman JS, Ono K, Stevenson DA, Yang X, Elefteriou F (2011). Mice lacking Nf1 in osteochondroprogenitor cells display skeletal dysplasia similar to patients with neurofibromatosis type I. Hum Mol Genet.

[30] DeClue JE, Cohen BD, Lowy DR (1991). Identification and characterization of the neurofibromatosis type 1 protein product. Proc Natl Acad Sci U S A.

[31] Basu TN, Gutmann DH, Fletcher JA, Glover TW, Collins FS, Downward J (1992). Aberrant regulation of ras proteins in malignant tumour cells from type 1 neurofibromatosis patients. Nature..

[32] Stevenson DA, Little D, Armstrong L, Crawford AH, Eastwood D, Friedman JM (2013). Approaches to treating NF1 tibial pseudarthrosis: consensus from the Children's Tumor Foundation NF1 bone abnormalities consortium. J Pediatr Orthop.

[33] Cho TJ, Seo JB, Lee HR, Yoo WJ, Chung CY, Choi IH (2008). Biologic characteristics of fibrous hamartoma from congenital pseudarthrosis of the tibia associated with neurofibromatosis type 1. J Bone Joint Surg Am.

[34] Khan T, Joseph B (2013). Controversies in the management of congenital pseudarthrosis of the tibia and fibula. Bone Joint J.

[35] O'Donnell C, Foster J, Mooney R, Beebe C, Donaldson N, Heare T (2017). Congenital Pseudarthrosis of the tibia. JBJS Rev.

[36] Diaz-Solano D, Wittig O, Mota JD, Cardier JE (2015). Isolation and characterization of multipotential mesenchymal stromal cells from congenital Pseudoarthrosis of the tibia: case report. Anat Rec (Hoboken).

[37] Paria N, Cho TJ, Choi IH, Kamiya N, Kayembe K, Mao R (2014). Neurofibromin deficiency-associated transcriptional dysregulation suggests a novel therapy for tibial pseudoarthrosis in NF1. J Bone Mineral Res.

[38] Evans DG, Bowers N, Burkitt-Wright E, Miles E, Garg S, Scott-Kitching V (2016). Comprehensive RNA analysis of the NF1 gene in classically affected NF1 affected individuals meeting NIH criteria has high sensitivity and mutation negative testing is reassuring in isolated cases with pigmentary features only. EBioMedicine..

[39] Ruggieri M, Huson SM (2001). The clinical and diagnostic implications of mosaicism in the neurofibromatoses. Neurology..

[40] Summerer A, Schafer E, Mautner VF, Messiaen L, Cooper DN, Kehrer-Sawatzki H (2019). Ultra-deep amplicon sequencing indicates absence of low-grade mosaicism with normal cells in patients with type-1 NF1 deletions. Hum Genet.

[41] Stevenson DA, Zhou H, Ashrafi S, Messiaen LM, Carey JC, D'Astous JL (2006). Double inactivation of NF1 in tibial pseudarthrosis. Am J Hum Genet.

[42] Lee SM, Choi IH, Lee DY, Lee HR, Park MS, Yoo WJ (2012). Is double inactivation of the Nf1 gene responsible for the development of congenital pseudarthrosis of the tibia associated with NF1?. J Orthop Res.

[43] McCaffrey M, Letts M, Carpenter B, Kabir A, Davidson D, Seip J (2003). Osteofibrous dysplasia: a review of the literature and presentation of an additional 3 cases. Am J Orthop (Belle Mead, NJ).

[44] Westacott D, Kannu P, Stimec J, Hopyan S, Howard A. Osteofibrous dysplasia of the tibia in children: outcome without resection. J Pediatr Orthop. 2017. 10.1097/BPO.0000000000001116 31393304

[45] Jung JY, Jee WH, Hong SH, Kang HS, Chung HW, Ryu KN (2014). MR findings of the osteofibrous dysplasia. Korean J Radiol.

[46] Park YK, Unni KK, McLeod RA, Pritchard DJ (1993). Osteofibrous dysplasia: clinicopathologic study of 80 cases. Hum Pathol.

[47] Mahnken AH, Staatz G, Hermanns B, Gunther RW, Weber M (2001). Congenital pseudarthrosis of the tibia in pediatric patients: MR imaging. AJR Am J Roentgenol.

[48] Neurofibromatosis (1988). National Institutes of Health Consensus Development Conference. Conference statement. Arch Neurol.

[49] Li H, Durbin R (2010). Fast and accurate long-read alignment with burrows-wheeler transform. Bioinformatics..

[50] Li H, Handsaker B, Wysoker A, Fennell T, Ruan J, Homer N (2009). The sequence alignment/map format and SAMtools. Bioinformatics..

[51] DePristo MA, Banks E, Poplin R, Garimella KV, Maguire JR, Hartl C (2011). A framework for variation discovery and genotyping using next-generation DNA sequencing data. Nat Genet.

[52] Wang K, Li M, Hakonarson H (2010). ANNOVAR: functional annotation of genetic variants from high-throughput sequencing data. Nucleic Acids Res.

[53] Li Q, Wang K (2017). InterVar: clinical interpretation of genetic variants by the 2015 ACMG-AMP guidelines. Am J Hum Genet.

[54] Yang H, Robinson PN, Wang K (2015). Phenolyzer: phenotype-based prioritization of candidate genes for human diseases. Nat Methods.

