# Supplementary figures and images for: Identification and characterization of NF1 and non-NF1 congenital pseudarthrosis of the tibia based on germline NF1 variants: genetic and clinical analysis of 75 patients
Source: Orphanet J Rare Dis. 2019 Sep 18;14:221. doi: 10.1186/s13023-019-1196-0 (PMC6751843; doi:10.1186/s13023-019-1196-0)

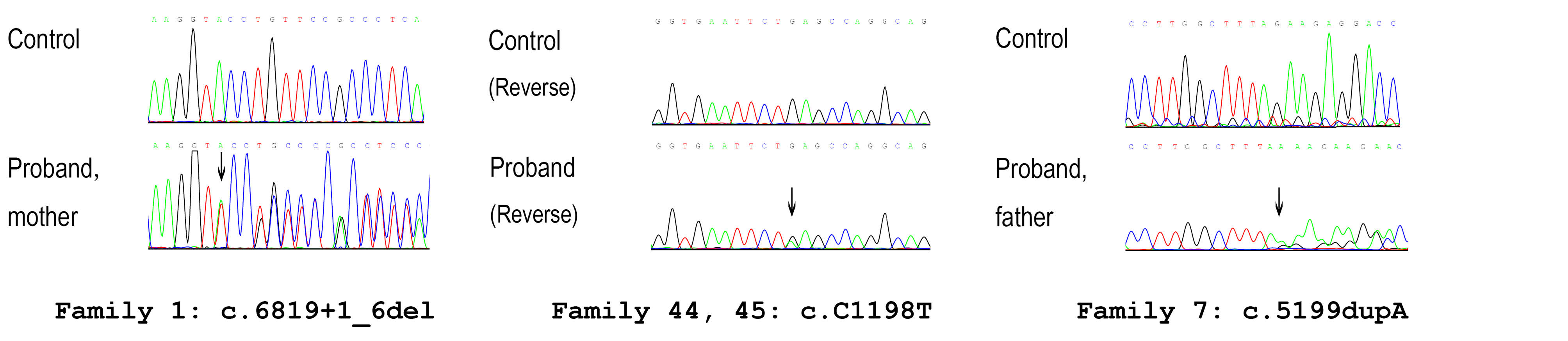

Supplement: Supplementary file 1 — Figure S1. Sequencing profile of identified variants in trios by Sanger sequencing. All 41 trios had performed Sanger sequencing and this figure shows three of them. “A” in sample ID represents probands, “B” represents the proband’s father, “C” represents the proband’s mother. (TIF 560 kb) [file 13023_2019_1196_MOESM1_ESM.tif]

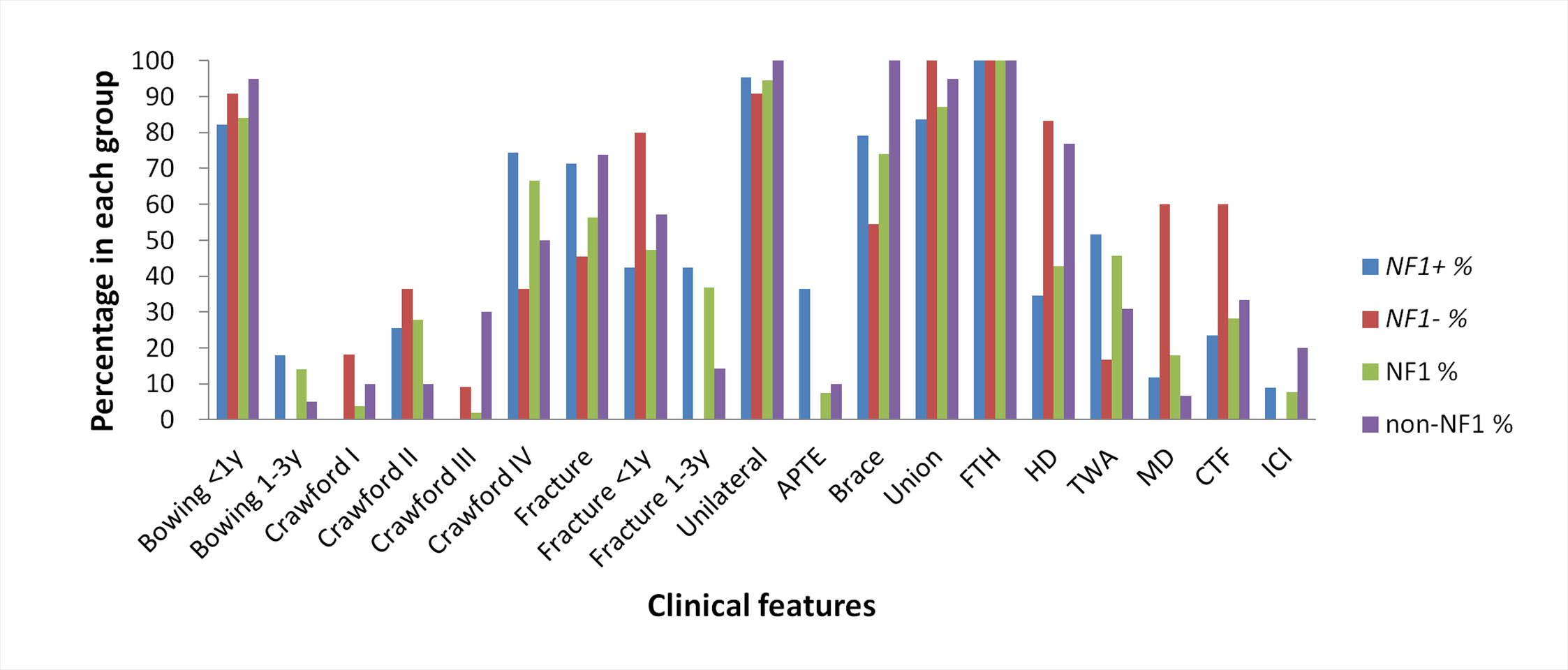

Supplement: Supplementary file 2 — Figure S2. Box plot of percentage of clinical features presented in four groups of CPT patients: NF1, non-NF1, NF1+ and NF1 − . (TIF 1261 kb) [file 13023_2019_1196_MOESM2_ESM.tif]

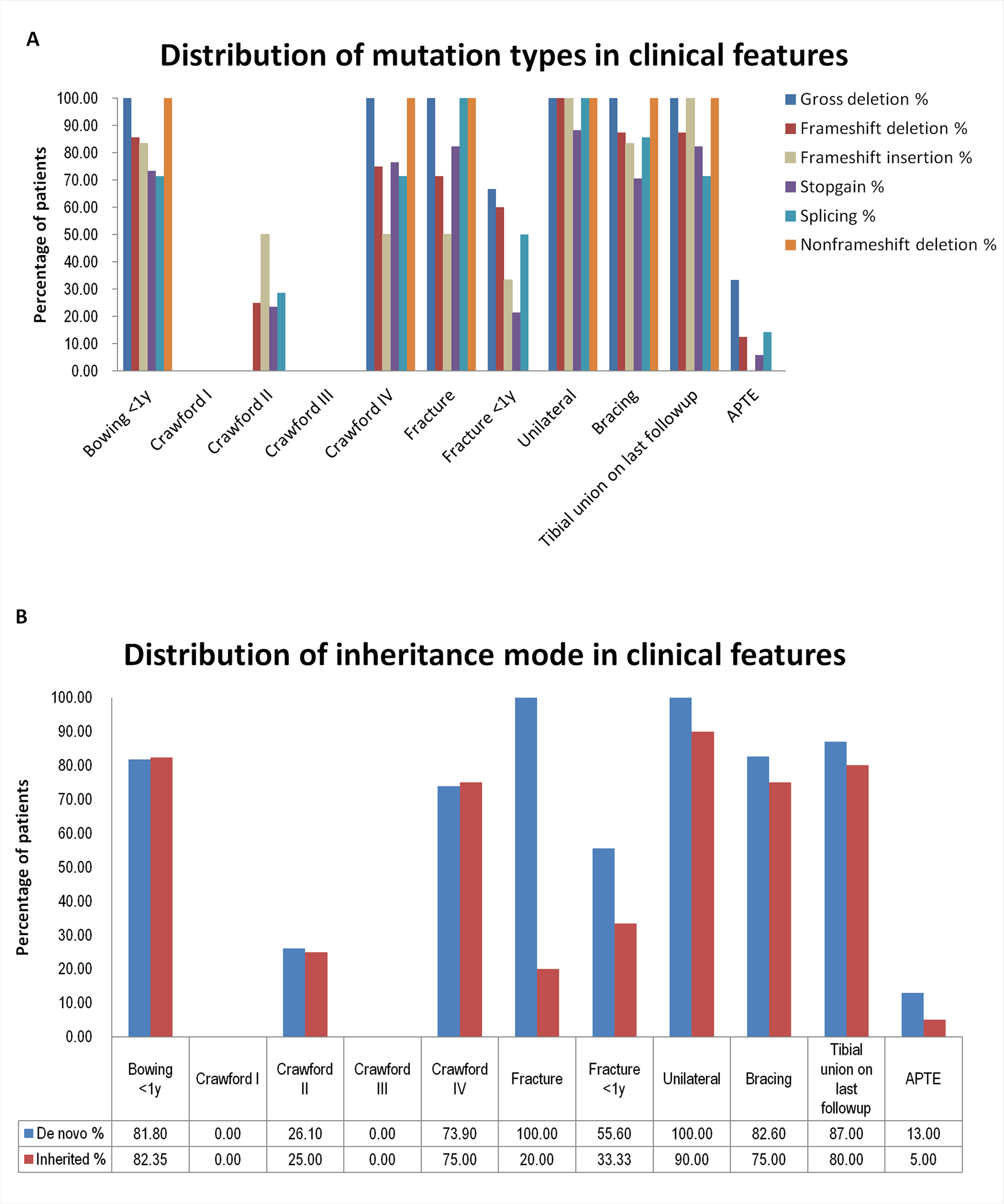

Supplement: Supplementary file 3 — Figure S3. Distribution of exonic functions and inheritance mode of variants in clinical features. A. Distribution of exonic functions against clinical features. No significant p-value of Fisher’ test was found in each feature. B. Distribution of inheritance mode against clinical features. Fracture shows a significant difference with p-value = 4.2E-05 (Fisher’s test). (TIF 2734 kb) [file 13023_2019_1196_MOESM3_ESM.tif]
